# Effects of mineral trioxide aggregate, calcium hydroxide, biodentine and Emdogain on osteogenesis, Odontogenesis, angiogenesis and cell viability of dental pulp stem cells

**DOI:** 10.1186/s12903-019-0827-0

**Published:** 2019-07-02

**Authors:** Abdel-Rahman Youssef, Ramy Emara, Mohiuddin M. Taher, Faisal A. Al-Allaf, Majed Almalki, Mazen A. Almasri, Shahid S. Siddiqui

**Affiliations:** 1 Department of Basic and Clinical Oral Sciences, Faculty of Dentistry, Umm Al-Qura University, Makkah, Kingdom of Saudi Arabia; 2 Department of Microbiology, Faculty of Medicine, Suez Canal University, Ismailia, Egypt; 3 Department of Restorative dentistry, Faculty of Dentistry, Umm Al-Qura University, Makkah, Kingdom of Saudi Arabia; 4 Department of Medical Genetics, Umm-Al-Qura University, Makkah, Kingdom of Saudi Arabia; 5 Science and Technology Unit Umm-Al-Qura University, Makkah, Kingdom of Saudi Arabia; 6 Oral Maxillofacial Surgery Department, Faculty of Dentistry, King Abdulaziz University, Jeddah, Kingdom of Saudi Arabia

**Keywords:** Dental pulp stem cells, Osteogenesis, Cytotoxicity, MTA, Ca(OH)_2_, Biodentin, Emdogain

## Abstract

**Background:**

Vital pulp therapy preserves and maintains the integrity and the health of dental pulp tissue that has been injured by trauma, caries or restorative procedures. The enhancement of cells viability and formation of reparative dentine and new blood vessels are vital determinants of the success of direct pulp capping. Therefore, the aims of this study was to evaluate and compare the in vitro osteogenic, odontogenic and angiogenic effects of mineral trioxide aggregate (MTA), calcium hydroxide [Ca(OH)_2_], Biodentine and Emdogain on dental pulp stem cells (DPSCs) and examine the effects of the tested materials on cell viability.

**Methods:**

DPSCs were treated with MTA, Ca(OH)_2_, Biodentine or Emdogain. Untreated cells were used as control. The cell viability was measured by MTT assay on day 3. Real-Time PCR with SYBR green was used to quantify the gene expression levels of osteogenic markers (alkaline phosphatase and osteopontin), odontogenic marker (dentin sialophosphoprotein) and angiogenic factor (vascular endothelial growth factor) on day 7 and day 14.

**Results:**

All capping materials showed variable cytotoxicity against DPSCs (77% for Emdogain, 53% for MTA, 26% for Biodentine and 16% for Ca(OH)_2_ compared to control (*P* value < 0.0001). Osteopontin (OPN) and dentin sialophosphoprotein (DSPP) gene expression was increased by all four materials. However, alkaline phosphatase (ALP) was upregulated by all materials except Emdogain. Vascular endothelial growth factor (VEGF) expression was upregulated by all four tested materials except Ca(OH)_2_.

**Conclusions:**

Our results suggest MTA, Biodentine and Emdogain exhibit similar attributes and may score better than Ca(OH)_2_. Emdogain could be a promising alternative to MTA and Biodentine in enhancing pulp repair capacity following dental pulp injury. However, further future research is required to assess the clinical outcomes and compare it with the in vitro findings.

## Background

Regenerative medicine is the process of restoring or regenerating human cells, tissues or organs for therapeutic applications [40]. Teeth are natural source of stem cells which are capable of self-renewal and multi-lineage differentiation into odontoblasts, osteoblasts, neurons, and endothelial cells [20, 53]. Dental stem cells are isolated from dental pulp, periodontal ligament and apical papilla [63].

The use of dental pulp stem cells (DPSCs) in research to understand the various mechanisms, gained momentum in many dental research laboratories. The method of DPSCs isolation was first reported in 2000 by Gronthos et al. [25]. DPSCs are characterized by expression of common differentiation markers including CD29, CD44, CD73, CD90, CD105, CD146 and absence of CD14, CD34, CD45 [54].

Vital pulp therapy maintains dental pulp vitality and preserves teeth [32]. Exposed vital pulp is treated by pulpotomy and direct pulp capping that induces formation of reparative dentine. Although DPSCs isolated from inflamed pulp have altered stem cell properties, they hold the potential to regenerate tissues [4]. The pulp capping materials form a protective layer over the nearly exposed or exposed vital pulp in direct and indirect pulp capping or in pulpotomy procedures [50]. Ideal pulp capping materials should be safe with good sealing ability and promote differentiation and proliferation of DPSCs [6, 44].

Carious lesion and/or physical injury start inflammation of the dental pulp. Mild or moderate inflammation induces pulp regeneration while severe and/or chronic inflammation damage the pulp. The materials used for pulp capping can modulate the balance between tissue inflammation and regeneration [23]. It has been shown that Calcium-silicate based cements extracts (TheraCal and Biodentine) induced injured and lipoteichoic acid -stimulated pulp fibroblasts to produce vascular endothelial growth factor (VEGF) and IL-6 [22]. However, TheraCal decreased pulp fibroblast proliferation and induced proinflammatory IL-8 secretion by pulp fibroblasts [29]. Biodentine was able to shift the balance from inflammation towards regeneration by decreasing TNF-induced Transient Receptor Potential Ankyrin 1 (TRPA1) expression and its functional activity [19].

It is important to study the properties of different capping materials in the context of cell viability of regenerating cells, transcriptional profile and their ability to affect differentiation process. Calcium Hydroxide [Ca(OH)_2_] is commonly used for direct pulp capping with sufficient biological responses [37]. However, the main disadvantages of Ca(OH)_2_ are weak cohesive strength, marginal leakage and inadequate antibacterial effect [2].

Mineral trioxide aggregate (MTA) was discovered by Torabinejad in 1993 [34]. MTA is well established pulp capping material because it has good biocompatibility, antimicrobial and sealing ability [48]. MTA can induce proliferation, and migration of human bone marrow-derived mesenchymal stem in vitro [16]. Clinically, MTA provided a biocompatible and long-term effective seal for root perforations [42]. MTA demonstrated superior hard tissue formation with less pulp inflammation compared with Ca(OH)_2_ [43]. However, the main drawbacks of MTA include long setting time, difficulty in removal after setting and its application must be in an area free from any infections [8, 10, 14, 15, 17].

Biodentine, a calcium silicate-based product, is introduced to the market in 2009. Biodentine is used for endodontic repair and pulp capping [38] and has good biocompatibility with DPSCs [9].

Enamel matrix derivative (EMD, Emdogain) is composed of a mixture of hydrophobic enamel matrix proteins derived from 6-month-old porcine tooth buds containing amelogenin, enamelin, tuftelin, amelin, and ameloblastin [11, 52], in a propylene glycol alginate (PGA). PGA has an antimicrobial activity [47] and enhances the regenerative potential of Emdogain [27]. In addition, Emdogain is shown to induce reparative dentin and can be used as a biologically active pulp-dressing agent [45, 46].

It is important to develop more effective pulp capping materials or at least improve the traditional ones [28, 62]. Several studies have investigated the effect of pulp capping materials on human adult dental pulp stem cells, but data is still scarce regarding their direct effects on dental pulp, and the differentiation mechanisms of DPSCs in response to these biomaterials. Here we have examined the hypothesis that the pulp capping materials MTA, Ca(OH)_2_, Biodentine and Emdogain have different cytotoxic, osteogenic and angiogenic effects on DPSCs. Our objectives were to compare the influence of the tested materials on the viability of DPSCs and emphasize their effectiveness in stimulating the reparative potential of dental pulp stem cells.

## Methods

### Experimental design

Dental pulp was extirpated and DPSCs were isolated and characterized by flowcytometry. DPSCs were treated with MTA, Ca(OH)_2_, Biodentine or Emdogain. Untreated cells were used as the control group. The cell viability of DPSCs was assessed using MTT assay on day 3. Osteogenic differentiation of DPSC was induced by osteogenic induction medium and gene expression of osteogenic markers [alkaline phosphatase (ALP) and osteopontin (OPN)], odontogenic marker [dentin sialophosphoprotein (DSPP)] and angiogenic factor (VEGF) were measured by Real-Time PCR based on SYBR green method on day 7 and 14.

### Dental materials

ProRoot MTA (*Dentsply Tulsa* Dental Specialties, Johnson City, *TN*, USA), calcium hydroxide (*Dycal*, DENTSPLY Caulk, USA) and Biodentine (Septodont, USA) were prepared according to manufacturer instructions and placed at the bottom of 6-well tissue culture plates, dried under laminar flow for 48 h at room temperature. Emdogain Gel 30 mg/ml (BIORA AB/Straumann, Switzerland) was diluted with alpha-modified Eagle medium (α-MEM) to a final working concentration of 100 μg/ml.

### Cell culture

Human adult third molars were collected from patients at Umm Al-Qura University teaching dental hospital after obtaining written informed consent and approval of ethical committee of the Faculty of Dentistry, Umm Al-Qura University. The teeth were collected in PBS and cut around the cementum-enamel junction to expose the pulp chamber. The pulp tissue was separated from the crown and root, and then digested in a solution of 3 mg/ml collagenase type I and 4 mg/ml dispase (Sigma, USA) for 1 h at 37 °C. The cells were gown in α-MEM growth medium (UFC Biotech, KSA) containing 10% fetal bovine serum (FBS) and 100 U/ml penicillin, 100 μg/ml streptomycin (HyClone, Thermo Fisher Scientific, USA) and incubated at 37 °C in 5% CO2.

### Flow cytometric surface marker expression analysis in human DPSCs

DPSCs cultured in α-MEM medium were analyzed for CD90 and CD45 cell surface antigens expression. Fluorescein isothiocyanate (FITC) - conjugated mouse anti-human CD90 and phycoerythrin (PE) conjugated mouse anti-human CD45 (BD Biosciences, USA) were utilized. DPSCs were detached from the plate using 0.25% trypsin with 1 mM EDTA (Gibco, Thermo Fisher Scientific, USA) and 50 μl of cell suspension (100,000 cells) was mixed with 5 μl of the corresponding antibody. After incubation for 30 min in the dark, the cells were washed and then acquired by flow cytometry (CYTOMICS FC 500 Flow Cytometer, Beckman Coulter, USA) and *analyzed* by *Cyflogic* v.1.2.1 software.

### Cell viability assay

The effect of pulp capping materials on cell viability of DPSCs was assessed using MTT (3-(4,5-dimethylthiazol-2-yl)-2,5-diphenyltetrazolium bromide) tetrazolium reduction assay according to manufacturer instructions (Abcam, UK). MTT penetrates viable eukaryotic cells due to its lipophilic side groups and positive net charge and reduced to water-insoluble formazan. The amount of formazan dye formed directly correlates to the number of metabolically active cells [49]. At 70% confluency, DPSCs were suspended in α-MEM growth medium and seeded at 5 × 10^4^ cells/well and incubated with MTA, Ca(OH)_2_, Biodentine or Emdogain in 24-well plates containing a final volume of 900 μl/well. Untreated cells were used as the control group. The cells were incubated at 37 **°**C in 5% CO_2_ for 3 days. On day 3, 100 μl MTT stock solution (5 mg/ml) was added to attached DPSCs in each well to achieve a final concentration of 0.5 mg/ml and incubated for 3 h at 37 °C. At the end of the incubation period the medium was removed and DMSO: isopropanol [1] solvent solution was added to dissolve formazan crystals. The solution was transferred to 96-well plate at 100 μl/well and optical density was read at 570 nm by a spectrophotometric Microplate Reader (SpectroStar Nano, BMG Lab).

### Osteogenic differentiation

DPSCs at the third passage were cultured in α-MEM growth medium at 2 × 10^5^/well into 6-well plates (Costar, Corning Life Sciences, USA). At 70% confluency, DPSCs were treated with the capping materials or left untreated (control). Osteogenic differentiation was induced by replacing the growth culture medium by osteogenic induction medium prepared as described previously [58]. Osteogenic medium was composed of α-MEM supplemented with 10% FBS, 100 IU/ml penicillin, 100 μg/ml streptomycin, 50 μg/ml L-ascorbic acid, 10 mM β -glycerophosphate, 10 nM calcitriol (1 α,25-dihydroxyvitamin D3) and 10 nM dexamethasone (all from Sigma. USA).

### Semi-quantitative real-time PCR

DPSCs were incubated in osteogenic medium in presence of MTA, Ca(OH)_2_, Biodentine or Emdogain. Untreated cells were used as the control group. After 7 and 14 days of treatment, DPSCs were rinsed three times in PBS and TRIzol Reagent (Invitrogen, Thermo Fisher Scientific, USA) was added directly to the attached cell to lyse them. After solubilization, chloroform was added and the upper phase containing the RNA was separated to which equal volume 70% ethanol was added. Subsequently binding, washing, and elution were performed using PureLink RNA mini kit according to the manufacturer’s protocol (Invitrogen, Thermo Fisher Scientific, USA). cDNA synthesis was performed by using high capacity cDNA reverse transcription kit according to manufacturer’s instruction (Applied Biosystems, Thermo Fisher Scientific, USA). 1 μg total RNA was used in reverse transcriptase reaction with reverse transcriptase, dNTPs, RNase inhibitor in a total volume of 20 μl. Semi-quantitative Real-Time PCR was performed using SYBR green (PowerUp SYBR Green Master Mix, Thermo Fisher Scientific, USA) and universal master mix in 20 μl volume. The Real-Time PCR was performed using the ABI 7500 Fast instrument (Applied Biosystems). The reaction condition comprised stage1: 50 °C for 2 min, 95 °C for 10 min 1 cycle; then stage 2: 95 °C 10 s. 60 °C for 1 min 40 cycles. Forward and Reverse Primers for genes examined are listed in Table 1. For quantification, we used the delta/delta calculation method (2^-ΔΔCT^ method) as described previously [61]. The Glyceraldehyde-3-phosphate dehydrogenase (GAPDH) gene was used as an internal control gene in all PCR experiments.

**Table 1:** Real-Time PCR Primers used for gene expression analysis

Gene	Primer sequence (5′-3′)	Ref
VEGF	F: 5’TGACAGGGAAGAGGAGGAGA-3′	[56]
	R: 5’CGTCTGACCTGGGGTAGAGA-3′	
ALP	F:5′ -ATGGGATGGGTGTCTCCACA-3′	[59]
	R:5′ -CCACGAAGGGGAACTTGTC-3′	
DSPP	F:5′-TTTGGGCAGTAGCATGGGC-3’	[59]
	R:5′- CCATCTTGGGTATTCTCTTGCCT-3’	
OPN	F:5′- CTCCATTGACTCGAACGACTC-3’	[59]
	R:5′-CAGGTCTGCGAAACTTCTTAGAT-3’	
GAPDH	F: 5′ - ATGGGGAAGGTGAAGGTCG-3’	[59]
	R: 5′- GGGGTCATTGATGGCAACAATA-3’	

### Statistical analysis

We compared the percentages of DPSCs viability in presence of pulp capping materials to untreated control using one-way analysis of variance (ANOVA). GraphPad Prism 7 (GraphPad Software, Inc., San Diego, CA) is used for Statistical analyses. The cell viability experiments were performed in triplicate and the results are expressed as mean ± standard error of the mean (SEM) and differences were significant if a *P* value is < 0.05.

## Results

### Characterization of DPSCs

**Fig. 1:**
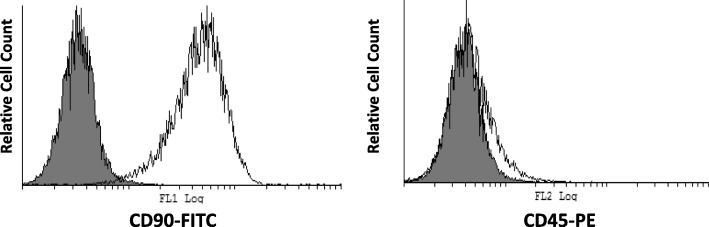
Surface marker expression of dental pulp stem cells by flow cytometry. Histogram representing the cell count (Y-axis) and fluorescence intensity on X-axis. Histogram overlays of unstained control cells (filled histogram) and cells stained with antibodies against the surface protein CD90-FITC and CD45-PE (empty histogram). It shows a positive peak shift for MSC marker CD90 and negative for hematopoietic lineage marker CD45. FITC: fluorescein isothiocyanate, PE: phycoerythrin

DPSCs were cultured in α-MEM growth medium and characterized by flow cytometry using CD90 and CD45 surface markers. The DPSCs were strongly positive for Mesenchymal stem cells (MSC) marker CD90 and negative for hematopoietic lineage marker CD45 as shown in Fig. 1.

### Cell viability of DPSCs

**Fig. 2:**
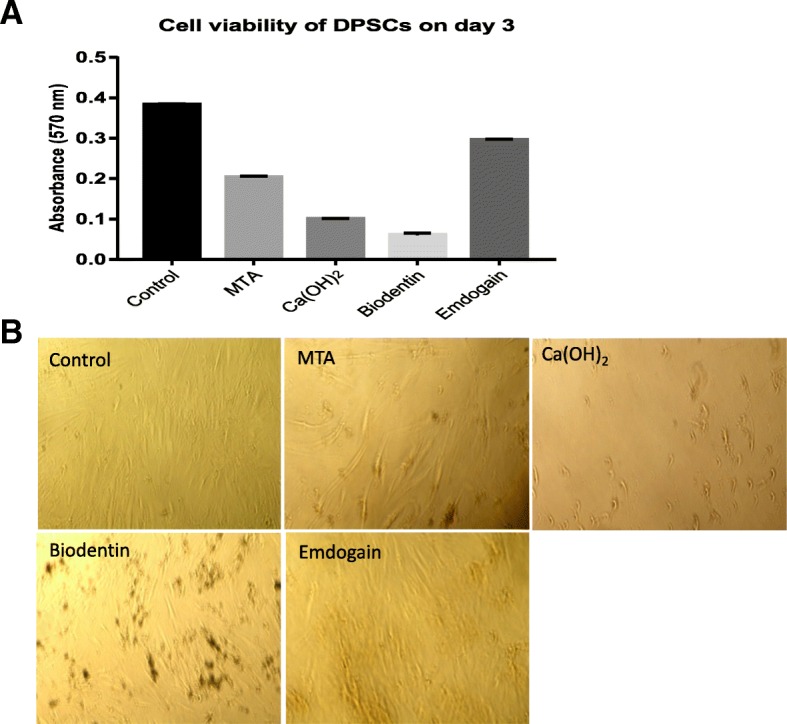
The effect of different pulp capping materials on cell viability of dental pulp stem cell after 3 days. (**a**) The cell viability was assessed by MTT assay and the percentage of stem cell viability compared to untreated control (100%). All capping materials MTA, Ca(OH)_2_, Biodentin and Emdogain showed variable cytotoxicity against DPSC compared to control (P value < 0.0001). (**b**) Morphological appearance of DPSC is shown after addition of different capping materials under light inverted microscopy. (magnification ×100). Microscopic pictures show reduction in the number of cells and morphological changes in treated cells especially with Ca(OH)_2_ compared to the untreated control. The experiment was performed in triplicate and the results are expressed as mean ± SEM

We used the MTT assay to compare the cytotoxic effect of MTA, Ca(OH)_2_, Biodentine and Emdogain on DPSC. The DPSCs were incubated in α-MEM growth medium containing 10% FBS in presence of the tested materials or left untreated (control). The cell viability of DPSCs was measured on day 3 using MTT assay (Fig. 2a). The percentage of stem cell viability was compared to control (100%). We have shown that all capping materials MTA, Ca(OH)_2_, Biodentine and Emdogain showed variable cytotoxicity against DPSCs compared to control (*P* value < 0.0001). Emdogain was the least cytotoxic to DPCS at 77% viability, followed by MTA at 53% whereas Ca(OH)_2_ and Biodentine showed significant cytotoxicity against DPCS at 26 and 16% viability respectively in comparison to control (P value < 0.0001). Microscopic pictures (Fig. 2b) show reduction in the number of cells and morphological changes in the cells treated with all capping materials especially `those treated with Ca(OH)_2_ compared to the untreated control.

### Gene expression of osteogenic, odontogenic and angiogenic markers

**Fig. 3:**
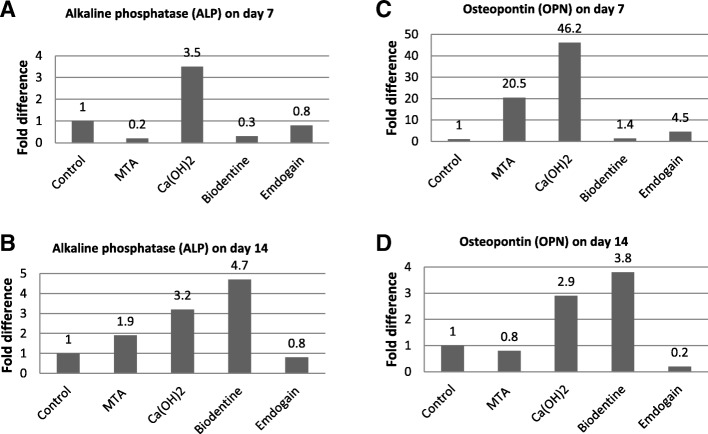
The effect of dental capping materials on expression of the osteogenic differentiation markers alkaline phosphatase (ALP) and osteopontin (OPN) by DPSCs. Fold change in gene expression of ALP and OPN at 7 and 14 days are shown in (**a**, **b**) and (**c**, **d**) respectively. The relative gene expression by real time PCR was normalized against the internal control gene (GAPDH) and relative to the untreated control. The control was set at 1 and the data were presented as the fold change in target gene expression relative to the untreated control

**Fig. 4:**
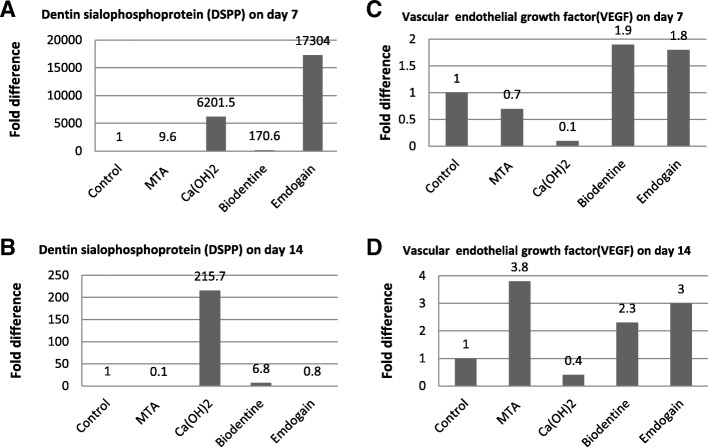
The effect of dental capping materials on expression of dentin sialophosphoprotein (DSPP) osteogenic differentiation marker and vascular endothelial growth factor (VEGF) by DPSCs. Fold change in gene expression of DSPP and VEGF at 7 and 14 days are shown in (**a**, **b**) and (**c**, **d**) respectively. The relative gene expression by real time PCR was normalized against the internal control gene (GAPDH) and relative to the untreated control. The control was set at 1 and the data were presented as the fold change in target gene expression relative to the untreated control

To investigate the effects of dental capping materials on expression of osteogenic, odontogenic and angiogenic markers, DPSCs were incubated in osteogenic medium in presence of MTA, Ca(OH)_2_, Biodentine or Emdogain. Untreated cells were used as control. Real Time-PCR was performed on day 7 and 14 (Figs. 3, 4). The relative gene expression by real time PCR was normalized against the internal control gene (GAPDH) and relative to the untreated control. The control was set at 1.0 and the data are presented as the fold change in target gene expression relative to the untreated control.

On day 7, Ca(OH)_2_ shows the greatest upregulation of ALP at 3.5-fold of control (Fig. 3A). However, on day 14, MTA, Ca(OH)_2_ and Biodentine increased expression of ALP compared to control (Fig. 3b). Regarding to OPN expression, MTA, Ca(OH)_2_ and Emdogain upregulated OPN expression on day 7 compared to control (Fig. 3c). However, on day 14, only Ca(OH)_2_ and Biodentine increased expression of OPN significantly in comparison to control (Fig. 3d). MTA, Ca(OH)_2_, Biodentine and Emdogain increased DSPP expression considerably on day 7 (Fig. 4a). However, on day 14, only Ca(OH)_2_ and Biodentine upregulated DSPP compared to control (Fig. 4b).

Angiogenic factor VEGF expression was upregulated by Biodentine and Emdogain on day 7 (Fig. 4c) whereas MTA, Biodentine and Emdogain increased VEGF expression on day 14 compared to control (Fig. 4d). However, Ca(OH)_2_ was the only capping material that reduced VEGF expression on day 7 and day 14.

## Discussion

Vital pulp therapy preserves pulpal injury, stimulates formation of dentine and maintain the dental pulp vitality. The capping material used for pulp therapy should be safe and induces pulp healing [32]. Analyzing the role of different biomaterials in the osteogenic gene expression and cell viability of stem cells is important for elucidating the molecular mechanisms underlying tissue engineering and regenerative medicine. Several studies investigated the expression of markers to osteogenesis after the application of pulp capping agents or cements to dental pulp cells [3, 35, 59]. The current study was designed to investigate the effects of MTA, Ca(OH)_2_, Biodentine and Emdogain on cell viability, and expression of osteogenesis, odontogenesis and angiogenesis markers by DPSCs.

Using stem cells specific markers in cytometry, we have examined the isolated human DPSCs. The cells were adherent to plastic plate and expressed CD90 stem cell marker and lack expression of the hematopoietic lineage marker CD45. These results fulfill the minimal criteria for defining multipotent mesenchymal stromal cells according to the International Society for Cellular Therapy [18]. Similar results of DPSCs characterization were reported by other studies [3, 31].

Cell viability assays are often used to determine the effects of test compound on cell proliferation or cytotoxicity. The most commonly used assays are the tetrazolium reduction, resazurin reduction and protease activity which measure general metabolism or an enzymatic activity of the viable cells and result in generating a signal that is proportional to the number of viable cells. Another frequently used assay is the luminogenic ATP assay in which the amount of ATP is directly proportional to the number of cells. ATP assay is the fastest and the most sensitive whereas the tetrazolium or resazurin reduction assays offer cheaper alternative with adequate performance. The most commonly used compounds in Tetrazolium reduction assays include MTS, XTT and WST-1 which do not penetrate cells and viable cell-penetrating MTT [51].

In this study, we used MTT assay to test the cytotoxic effect of pulp capping materials on DPSCs. Our results show that all capping materials MTA, Ca(OH)_2_, Biodentine and Emdogain showed variable cytotoxicity against DPSCs compared to control when no addition was made in the cell growth medium. Our data are in agreement with Bortoluzzi et al. [9] who have found that cell viability was significantly affected in the presence of MTA and Biodentine, or their released eluents. In addition, the cell viability was reduced in presence of MTA [33] and Ca(OH)_2_ [26]. Similarly, TheraCal decreased pulp fibroblast proliferation by pulp fibroblasts [29]. Moreover, other studies [1, 35, 60] have showed that DPSCs viability on MTA was significantly lower when compared with that of cells on Biodentine in the first seven days.

Contradictory results were observed by other studies. Luo et al. [36] observed that proliferation of human DPSCs was increased by Biodentine at 2 mg/ml and 0.2 mg/ml and decreased at 20 mg/ml suggesting that the capping material concentration might affect cell viability. In another study, Wang et al. [59] have found that Emdogain enhanced proliferation of DPSCs in osteogenic induction medium and Araújo et al. [5] have shown that the cell viability of stem cells from human exfoliated deciduous teeth was similar or higher than to control after treatment with MTA, Ca(OH)_2_ or Biodentine. The mechanisms of observed cytotoxicity are not clearly understood. It was suggested that initial release of calcium-ions, ionic activities, presence of toxic components or pH changes may affect the behavior of the cells [21, 41]. Results shown here may be applied in future studies to modify the surface of the materials to promote better cell viability and sprouting of cells in tissue regeneration protocols.

Bone formation (osteogenesis) play a crucial role in the dental tissue regeneration [25, 39, 46]. Osteogenic and odontogenic differentiation of DPSCs is commonly assessed by expression of related markers and play a key role during initial odontoblastic differentiation and late dentin mineralization [13]. Our results have shown that gene expression of osteogenic and odontogenic markers in DPSCs were upregulated by MTA, Ca(OH)_2_, Biodentine and Emdogain. Similar results were reported by other studies, for *ex.,* osteogenesis markers were upregulated by Emdogain [26, 30, 59], MTA and Biodentine [9]. There is a mutual relationship between the decrease in proliferation and the consequent gene upregulation associated with matrix maturation and mineralization suggesting that the extracellular matrix contributes to both the shut-down of proliferation and development of the osteoblast phenotype [57].

Vasculogenesis is the process mediating development of vascular system and angiogenesis. The growth of blood vessels emanating from existing vasculature are critical in tissue regeneration. In endodontic regeneration various studies have examined the effect of biomaterials on vascularization and angiogenesis [7, 24, 25]. Several angiogenesis markers such as VEGF have been investigated. VEGF family of proteins controls both vascularization and angiogenesis. VEGF stimulates the endothelial cells close to microvessels to proliferate, migrate and change their pattern of gene expression [12]. In the current study, we have demonstrated that Emdogain and Biodentine increased the expression of VEGF on day 7, whereas MTA, Biodentine and Emdogain up-regulated VEGF on day 14 compared to control but Ca(OH)_2_ was the only capping material that did not upregulate VEGF on either the day 7 or day 14. These data suggest that Ca(OH)_2_ is not conducive to angiogenesis and acidogenesis, whereas MTA, Biodentine and Emdogain induce VEGF expression, thereby facilitating tissue regeneration. Attempts have been made in examining the angiogenesis in the presence of biomaterials [39, 55]. However, results presented here are the first investigation on human DPSCs angiogenesis when exposed to four different biomaterials (MTA, Ca(OH)_2_, Biodentine and Emdogain), and allow a unique comparison that has not been reported previously.

## Conclusions

Our results indicated that all tested materials affected cell viability of dental pulp cells and promoted in vitro pulp repair mechanisms via upregulation of odontogenic and angiogenic gene markers in varying degrees. Emdogain was the least toxic to the DPSCs compared to MTA, Ca(OH)_2_ and Biodentine. In case of osteogenic potential of DPSCs, gene expression of osteogenic and odontogenic markers was increased by all four tested materials. For the angiogenesis marker VEGF expression, we find that all tested materials elevated expression of the VEGF except for Ca(OH)_2_. Thus, our results suggest that considering human DPSCs cell viability and molecular expression of angiogenesis and osteogenesis markers, MTA, Biodentine and Emdogain are comparable and may score better than Ca(OH)_2_, as capping material. Collectively, our results indicated that Emdogain could be a promising alternative to MTA and Biodentine in enhancing pulp repair capacity following dental pulp injury.

This study has some potential limitations, which we do not assume to have significantly impacted on our findings. One of these limitations is that we have relied on the MTT assay to examine cell viability and not fully examined the cellular toxicity from apoptosis and necrotic cell death pathways, that remain to be studied. Further studies to assess the inflammatory mediators resulting from interaction of DPSCs with pulp capping materials may explain cytotoxicity of these materials. Additional studies are needed to assess the clinical outcomes and compare it with the in vitro findings.

## Data Availability

Available.

## Abbreviation

Ca(OH)_2_: Calcium hydroxide
ALP: Alkaline phosphatase
DPSCs: Dental pulp stem cells
DSPP: Dentin sialophosphoprotein
EMD,Emdogain: Enamel matrix derivative
FBS: Fetal bovine serum
FITC: Fluorescein isothiocyanate
GAPDH: Glyceraldehyde-3-phosphate dehydrogenase
MTA: Mineral Trioxide Aggregate
OPN: Osteopontin
PE: Phycoerythrin
VEGF: Vascular endothelial growth factor
α-MEM: Alpha -modified Eagle medium
